# Lawson Wilkins: portrait of a pioneer

**DOI:** 10.1186/1687-9856-2014-S1-I1

**Published:** 2014-05-28

**Authors:** John S Fuqua, Peter A Lee

**Affiliations:** 1 Indiana University School of Medicine, Indianapolis, IN, USA; 2 Penn State Milton S. Hershey Medical Center, Hershey, PA, USA

## Foreword

**Figure 1:**
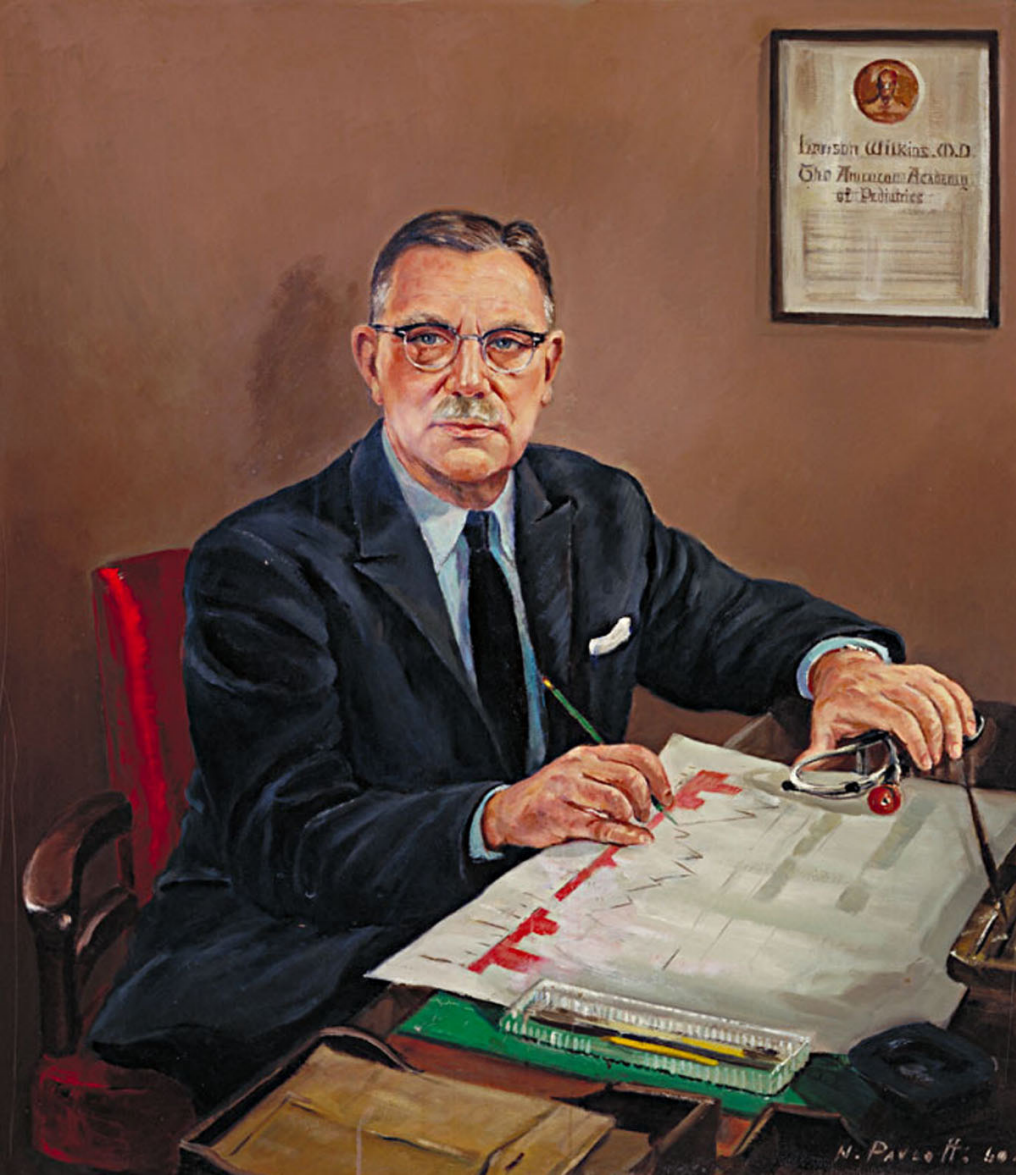
Lawson Wilkins portrait.

September 27, 2013 was the 50^th^ anniversary of Lawson Wilkins’ death. After reading the two articles by his daughter, Betsy McMaster, and his devoted protégée, Claude Migeon, we can’t help but wonder what Dr. Wilkins would have thought if he could see how we practice Pediatric Endocrinology today. Lawson Wilkins was born in 1894, and his father (also a physician) would take him along on his rounds in a horse and buggy. After serving in World War I and graduating from Johns Hopkins School of Medicine, Dr. Wilkins completed his pediatric residency at Yale and then settled down in his home town of Baltimore to practice general pediatrics. However, he quickly distinguished himself from other practitioners by his insatiable curiosity and careful attention to measurable physical parameters and the information they provided for diagnosis and clinical management. He became renowned for the meticulous graphs and charts that he hand-prepared as illustrated in the famous portrait. (Figure 1) As he studied growth and development of normal children and contrasted these with patterns in children with hypothyroidism, his interest in endocrinologic disorders increased. He developed a relationship with Dr. Edwards Park, Chief of Pediatrics at Hopkins. Dr. Park was eventually able to recruit Dr. Wilkins to a part time and then a full time faculty position. But much of his early work in hypothyroidism was conducted while he was working diligently in his pediatric practice. After giving up his practice in 1946, he devoted himself full time to endocrinology, resulting, with the help of his fellows and associates, in a rapid expansion of scientifically validated knowledge.

No doubt that Dr. Wilkins saw tremendous changes over the course of his medical career, perhaps the most important one being the development of antibiotics, which saved the lives of countless children. But Dr. Wilkins himself was the driver of changes in endocrine practice. In fact, while trying to learn from his predecessors about endocrinology in children, he ended up throwing an existing text of endocrine disease against the wall in frustration and decided to write his own based on his collection of painstakingly-developed case observations. This work eventually led to the first textbook of Pediatric Endocrinology and stimulated interest in the subspecialty around the world. But if he could have travelled forward in time to 2013, what would he think of where we are now?

He would certainly have been amazed by the technology. Measurement of hormones in his day relied on relatively primitive time and labor-intensive chromatographic and colorimetric techniques. Radioimmunoassays were perfected after his death, and platform-based immunoassays and tandem mass spectrometry were not even dreams. He might have been jealous of our ability to draw a blood sample and know the 17-hydroxyprogesterone or T4 concentration on the same day! As noted above, much of Dr. Wilkins’ early work in Pediatric Endocrinology was in the area of hypothyroidism, particularly congenital hypothyroidism, and we think he would be happy to see that universal newborn screening has essentially eliminated cretinism. Another area of focus was growth and its disorders. He lived to see the early use of pituitary-derived growth hormone in the treatment of growth hormone deficiency, and another of his protégés, Dr. Blizzard, was instrumental in the foundation and direction of the National Pituitary Agency. Our current detailed understanding of growth hormone secretion, physiology of the GH-IGF axis, and molecular genetics of growth disorders would have answered many of his questions but no doubt would have brought on new ones. Much of Dr. Wilkins’ academic career was spent in the investigation of adrenal disorders, puzzling out the now well-known steroidogenic pathways and devising treatment approaches for congenital adrenal hyperplasia. He probably would be gratified that the therapies he developed are not much different today, but might have been disappointed that even now CAH treatment is less than perfect.

There are also aspects of our specialty and medicine in general that we think would disappoint him. The Pediatric Endocrine Clinic at Johns Hopkins in the 1950s was an intellectually stimulating place. The Saturday Clinic was a venue that brought together a cohort of bright, curious physicians who, led by Dr. Wilkins, would brainstorm ideas about normal and abnormal physiology, new treatment approaches, and ideas to answer the many unknowns through clinical and basic research. Dr. Wilkins would regret the present-day outside influences pushing higher the number of patients to be seen in a limited time and the resulting lack of time afforded to critical thinking about our patients and their problems. Additionally, Dr. Wilkins was a premier physical diagnostician, and he was able to learn tremendously from studying the natural history of his patients. Today, he might have perceived a shift of focus from the patient’s overall clinical picture as illustrated by fundamental historical and physical examination findings to at times excessive reliance on laboratory test results and attention to minor lab abnormalities.

Overall, if Dr. Wilkins could see where we have come in the 50 years since his death, we think he would be very gratified to see the growth of the subspecialty that he, along with colleagues in Boston and in Europe, founded. In the US, the American Board of Pediatrics notes that there are now nearly 1500 board certified pediatric endocrinologists. He might also be surprised to learn that just over 50% of them are women, and this might cause him to reconsider his opinion that women should not be examining male genitalia!

It has been common for pediatric endocrinologists to figure out how many professional generations removed they were from Dr. Wilkins. Did you train with one of his protégés? With a protégé of a protégé? As the years have passed, however, the memory of Dr. Wilkins has begun to fade. Because of this, we hope you will read this collection of reminiscences and gain new insights into the birth of our profession under the guidance of a brilliant physician.

## Competing interests

The authors declare that they have no competing interests.

## Declarations

This article has been published as part of *International Journal of Pediatric Endocrinology* Volume 2014 Supplement 1, 2014: Remembering Doctor Lawson Wilkins: a pioneer of pediatric endocrinology. The full contents of the supplement are available online at http://www.ijpeonline.com/supplements/2014/S1.

